# Emanations

**Published:** 1874-07

**Authors:** 


### EMANATIONS.

PROBABLY everything has its odor, or scent, or emanation; something which it throws off from itself, agreeable or disagreeable, by which its presence is known; that emanation is corporeal, has weight, but is so near being imponderable that it can be breathed into the lungs or swallowed into the stomach to be absorbed, and entering into the circulation becomes a part of the volume of the blood to corrupt, to poison and to kill.

But, however delightful the odor of the rose or the pink may be, the air which it impregnates is not as healthful, as lifegiving as if that air had nothing but its natural ingredients—was pure.

The odor of carrion is so disgusting that we are instinctively driven away from it. Unhealthful odors generally make us aware of their presence by their disagreeableness; thus it is that we are prompted to put filth away from us, and keep our persons, and clothing, and dwellings neat, and tidy, and clean. But emanations, some of them the most deadly, are not always appreciable to the senses, and we breathe them and die, sometimes. A man goes to sleep in a close room, where charcoal is burning in an open pan, to keep himself warm; he becomes drowsy, goes to sleep, and dies before morning, without his having perceived that he was breathing anything else than the ordinary atmosphere; this was because the burning charcoal gave out, had an emanation coming from it, called "carbonic acid gas," which is deadly, if too large a proportion is found in the air breathed. More than one volume or bulk of carbonic acid gas in a thousand volumes of air is injurious to health. MIASM is an emanation from the decomposition of wood, leaves or other forms of decaying vegetable matter. The air may be full of it in the cool of an autumn evening; about sunrise it is perfectly delicious, and yet so pernicious as to engender fatal fevers and other forms of disease.

A person may sleep in a room covered with unvarnished green velvet, or gloss paper; or a child may play with green toys and be thrown into convulsions and die in a few days, or even hours, from the emanations of particles of arsenic contained in the green color; in neither case would any odor be perceptible.

Some persons coming into a room where powdered ipecac is handled, will, in a few minutes, begin to water at the nose and eyes, and have a copious discharge from both, like those having a cold in the head or catarrh.

Others are thrown into a violent commotion, or have other physical disturbances of the system if a dog comes into the room. Cases are given of persons who have "hay fever" at a time of year when the new mown hay gives out its perfume or emanation. Other cases are recorded where persons were thrown into asthmatic symptoms, painful difficulty in breathing, if a cat came into the room, whether the cat was seen or not; it is because of the emanations from the cat acting on an organization of peculiar character; it is not a mere notion, as ignorant or unsympathizing persons assert.

The odor of a rose has had the same effect on even distinguished persons. The scientific name of these peculiarities is "idiosyncrasy."

This whim of nature, as it may be called, is born with the person; it is an inherited quality. When these peculiarities are observed in young children they are deepened or aggravated if scolded or laughed at. A better plan is to sympathize with the child, and encourage him to strive against the misfortune, or live it down, by accustoming him to handle, or touch, or smell the article in question, first for a minute or two, or longer; if this is borne better and better perseverance may cure in time, or at least greatly modify the misfortune; if, however, the symptoms are aggravated, yield to the necessity and do the next best thing—keep out of the way the enemy of your peace.

